# Bleeding‐Related Perinatal Outcomes Following Hormone Replacement Cycle Frozen Embryo Transfer Compared With Non‐ART, Fresh Embryo Transfer, and Ovulatory‐Cycle Frozen Embryo Transfer: A Single‐Center Cohort Study

**DOI:** 10.1002/rmb2.70110

**Published:** 2026-10-29

**Authors:** Tatsuya Yoshihara, Yosuke Ono, Dai Miyashita, Maki Ogi, Kota Tanaka, Satoko Sasatsu, Eriko Ogasahara, Yasuhiko Okuda, Osamu Yoshino

**Affiliations:** ^1^ Department of Obstetrics and Gynecology University of Yamanashi Yamanashi Japan

**Keywords:** assisted reproductive technology, corpus luteum, frozen embryo transfer, hormone replacement cycle, placenta accreta spectrum, postpartum hemorrhage

## Abstract

**Purpose:**

Frozen embryo transfer (FET) is associated with increased maternal complications, but whether these risks are specific to the hormone replacement cycle (HRC‐FET) protocol or shared broadly across FET pregnancies remains unclear. This study compared perinatal outcomes among non‐ART, fresh embryo transfer, ovulatory‐cycle FET (OC‐FET), and HRC‐FET pregnancies.

**Methods:**

This was a single‐center retrospective cohort study of 5417 singleton deliveries (2017–2025), comprising 4427 non‐ART, 158 fresh embryo transfer, 208 OC‐FET, and 624 HRC‐FET pregnancies. Multivariable logistic regression, adjusted for maternal age, BMI, and parity, was used to calculate adjusted odds ratios (aORs), with non‐ART as the reference group.

**Results:**

Compared with non‐ART, HRC‐FET was associated with significantly increased risks of manual removal of the placenta (aOR 14.54), postpartum hemorrhage (aOR 2.33), and uterine atony (aOR 3.23). These risks remained significant after stratifying by mode of delivery. In contrast, fresh embryo transfer and OC‐FET showed risk profiles resembling non‐ART for most outcomes.

**Conclusion:**

Increased perinatal risk associated with ART is not uniform but concentrated primarily in HRC‐FET. Perinatal management after HRC‐FET should account for bleeding‐related complications.

## 1 Introduction

In recent years, the number of births conceived through assisted reproductive technology (ART) has increased [1]. Perinatal management of ART‐conceived pregnancies has therefore become an important issue in obstetric practice. Pregnancies conceived through ART have been associated with increased risks of several adverse maternal and perinatal outcomes compared with pregnancies conceived without ART [2, 3]. However, ART encompasses heterogeneous treatment modalities, and these risks may differ according to the specific treatment used [4].

Among ART procedures, frozen embryo transfer (FET) has become widely adopted and now plays a central role in contemporary ART practice [1]. However, pregnancies conceived via FET have been reported to be associated with an increased risk of maternal complications, including placenta accreta spectrum (PAS), postpartum hemorrhage (PPH), and hypertensive disorders of pregnancy (HDP), compared with naturally conceived pregnancies and those conceived via fresh embryo transfer [5, 6, 7]. Notably, FET pregnancies are themselves heterogeneous, and accumulating evidence suggests that these risks may differ according to the endometrial preparation protocol used. In FET, endometrial preparation is achieved primarily through one of two protocols: a hormone replacement cycle (HRC) FET or an ovulatory cycle (OC) FET. During an HRC, ovulation does not occur, and the corpus luteum is therefore not formed. Consequently, corpus luteum–derived vasoactive substances and the early pregnancy endocrine milieu are thought to differ from those in an OC [8, 9]. Previous studies comparing HRC and OC have reported an increased incidence of PAS and preeclampsia in HRC‐FET [5, 10]. However, evidence regarding protocol‐specific risks has largely been derived from comparisons between HRC‐FET and OC‐FET. It therefore remains unclear whether the adverse outcomes observed after ART and FET represent risks broadly shared across treatment modalities or are disproportionately associated with specific protocols, particularly HRC‐FET. Clarifying this issue would allow ART pregnancies to be evaluated according to treatment subgroup, rather than being uniformly regarded as high‐risk. Such clarification has considerable clinical significance, as it could inform management strategies tailored to the specific endometrial preparation protocol used. Furthermore, by comparing fresh embryo transfer and OC‐FET with non‐ART pregnancies, it becomes possible to determine whether these groups carry a perinatal risk comparable to that of HRC‐FET, or whether their risk more closely resembles that of non‐ART pregnancies.

In this study, we used a single‐center database to compare perinatal outcomes among four groups: non‐ART, fresh embryo transfer, OC‐FET, and HRC‐FET. We focused particularly on bleeding‐related outcomes, confirming the elevated risk previously associated with HRC‐FET while also examining whether fresh embryo transfer and OC‐FET show a similarly elevated bleeding‐related risk, or instead show a risk profile resembling that of non‐ART pregnancies.

## 2 Materials and Methods

### 2.1 Study Design and Participants

This was a single‐center retrospective cohort study of all deliveries at our institution between January 2017 and December 2025. Our institution is a university hospital designated as a regional perinatal medical center, and it receives referrals of high‐risk pregnancies. Multiple pregnancies were excluded, as were FET cases in which the endometrial preparation protocol was unknown, primarily because infertility treatment had been performed at another institution.

### 2.2 Group Classification

Participants were classified into four groups based on the mode of conception and embryo transfer protocol: non‐ART, fresh embryo transfer, OC‐FET, and HRC‐FET. The non‐ART group comprised pregnancies conceived without ART. The fresh embryo transfer group comprised pregnancies conceived via fresh embryo transfer. The OC‐FET group comprised pregnancies conceived via FET during either a natural or letrozole‐induced ovulatory cycle; these cycles were grouped together because both involve ovulation and corpus luteum formation. The HRC‐FET group comprised pregnancies conceived via FET during an HRC.

### 2.3 Patient Background Characteristics

The following patient background characteristics were collected: maternal age at delivery, pre‐pregnancy body mass index (BMI), parity (nulliparous or multiparous), and the presence of uterine myoma, adenomyosis, or endometriosis. To characterize differences in ART treatment background among the three ART groups (fresh embryo transfer, OC‐FET, and HRC‐FET), we compared the rate of intracytoplasmic sperm injection (ICSI), the blastocyst transfer rate, the number of embryos transferred (one or two), and the good‐quality embryo transfer rate. A good‐quality embryo was defined as a blastocyst in which neither the inner cell mass grade nor the trophectoderm grade was C; when two embryos were transferred, the transfer was classified as good‐quality if at least one embryo met this criterion. The good‐quality embryo transfer rate was calculated only among blastocyst transfers. A history of endometriosis or adenomyosis was recorded from patient self‐report, information in referral letters, or findings at our institution; no uniform diagnostic criterion (such as imaging, surgery, or pathology) was applied. Endometriosis, adenomyosis, and their combination (endometriosis or adenomyosis, with patients having both conditions counted once) were each tabulated.

### 2.4 Outcome Measures

The primary outcomes were bleeding‐related outcomes: manual removal of the placenta, PPH, blood loss ≥ 1500 mL, blood loss ≥ 2000 mL, and uterine atony. Manual removal of the placenta was defined as the actual performance of the procedure, as recorded in the delivery records. For vaginal delivery, it was performed when spontaneous placental separation did not occur during the third stage of labor; approximately 30 min was used as a general guide for waiting [11], but the final decision on whether and when to perform manual removal was left to the attending obstetrician. For cesarean delivery, it was performed when placental separation failed despite attempted delivery of the placenta with uterine massage. There was no standardized institutional protocol, and the decision was influenced by the amount of bleeding, suspicion of PAS, maternal hemodynamic status, and clinical judgment. Manual removal was occasionally performed earlier in cases of heavy bleeding without spontaneous separation. PPH was defined, based on the World Health Organization definition, as blood loss ≥ 500 mL for vaginal delivery and ≥ 1000 mL for cesarean delivery [12]. Uterine atony was defined as inadequate uterine contraction despite the routine oxytocin regimen used at our institution for both vaginal and cesarean deliveries (oxytocin 10 units in 500 mL of acetated Ringer's solution, administered at 250 mL/h), requiring additional treatment, such as intensified uterotonic therapy, bimanual uterine compression, intrauterine balloon tamponade, or uterine compression sutures, and diagnosed as uterine atony by the attending obstetrician. No blood loss threshold was included in the definition.

The secondary outcomes were cesarean delivery, emergency cesarean delivery, elective cesarean delivery, HDP, preeclampsia, superimposed preeclampsia, gestational hypertension (GH), gestational diabetes mellitus (GDM), placenta previa, placental abruption, premature rupture of membranes (PROM), meconium‐stained amniotic fluid, oligohydramnios, labor induction or augmentation, admission to the neonatal intensive care unit (NICU), preterm birth before 37 weeks of gestation, preterm birth before 34 weeks of gestation, low birth weight, very low birth weight, small for gestational age (SGA), large for gestational age (LGA), fetal growth restriction (FGR), low Apgar score at 1 and 5 min, and low umbilical artery pH. A low Apgar score was defined as < 7 points, and a low umbilical artery pH was defined as < 7.1. FGR was defined as an estimated fetal weight (EFW) ≤ −1.5 SD on at least one occasion during pregnancy, based on Japanese fetal biometric charts by gestational age [13]. SGA was defined as a birth weight below the 10th percentile for gestational age, sex, and birth order, using the gestational age–specific growth charts proposed by the Neonatology Committee of the Japan Pediatric Society [14]. LGA was defined, using the same charts, as a birth weight at or above the 90th percentile for gestational age, sex, and birth order [14]. Subtypes of HDP (GH, preeclampsia, and superimposed preeclampsia) were classified according to the criteria of the Japan Society for the Study of Hypertension in Pregnancy [15].

### 2.5 Statistical Analysis

Continuous variables are presented as medians with interquartile ranges and were compared among groups using the Kruskal–Wallis test. Categorical variables are presented as numbers with percentages and were compared among groups using the chi‐squared test.

For all primary and secondary outcomes, we first calculated crude odds ratios (OR) with 95% confidence intervals (CI) using univariable logistic regression. We then performed multivariable logistic regression adjusted for maternal age, pre‐pregnancy BMI, and parity, and calculated adjusted odds ratios (aOR) with 95% CI. For each analysis, cases with missing data for the outcome or any covariate included in the corresponding model were excluded. The non‐ART group served as the reference group. We additionally performed multivariable logistic regression, adjusted in the same manner, for all pairwise comparisons among the four groups (fresh embryo transfer, OC‐FET, HRC‐FET, and non‐ART; six comparisons in total).

To assess the influence of the mode of delivery, we also performed a stratified analysis by vaginal delivery and cesarean delivery, calculating aOR adjusted in the same manner within each stratum. A two‐sided *p*‐value < 0.05 was considered statistically significant for all analyses. For the primary bleeding‐related outcomes, results of all pairwise comparisons were additionally summarized using a compact letter display, in which groups not sharing the same letter differed significantly in pairwise comparisons. Given the exploratory nature of this study, no adjustment for multiple comparisons was performed. As a sensitivity analysis, we performed multivariable logistic regression for the primary bleeding‐related outcomes with endometriosis or adenomyosis added to the original model (maternal age, pre‐pregnancy BMI, and parity). All six pairwise comparisons were examined.

### 2.6 Ethical Considerations

This study was approved by the Ethics Committee of the University of Yamanashi (approval no. 3095). The requirement for written informed consent was waived because of the retrospective nature of the study, and an opt‐out approach was used in accordance with the ethics committee approval.

## 3 Results

### 3.1 Patient Characteristics

Of the 5744 deliveries during the study period, 122 multiple pregnancies and 205 FET pregnancies with an unknown endometrial preparation protocol were excluded, leaving 5417 cases for analysis. The number of cases in each group was as follows: 4427 in the non‐ART group, 158 in the fresh embryo transfer group, 208 in the OC‐FET group, and 624 in the HRC‐FET group.

Significant differences were observed among the four groups in maternal age at delivery, pre‐pregnancy BMI, nulliparity, and the presence of uterine myoma. Maternal age at delivery and the proportion of nulliparous women were higher in the fresh embryo transfer, OC‐FET, and HRC‐FET groups than in the non‐ART group. The prevalence of endometriosis or adenomyosis differed significantly among the groups (non‐ART, 1.5%; fresh embryo transfer, 4.4%; OC‐FET, 8.2%; HRC‐FET, 4.3%; *p* < 0.001), mainly because of the difference in endometriosis (Table 1).

**TABLE 1:** Comparison of background characteristics among the non‐ART, fresh embryo transfer, OC‐FET, and HRC‐FET groups.

Characteristic	Non‐ART (*n* = 4427)	Fresh ET (*n* = 158)	OC‐FET (*n* = 208)	HRC‐FET (*n* = 624)	*p*
Maternal age at delivery (years)	32.0 (29.0–36.0)	36.0 (33.0–38.0)	36.0 (33.0–39.0)	35.0 (32.0–38.0)	< 0.001
Pre‐pregnancy BMI	21.0 (19.2–23.5)	20.8 (19.2–22.8)	20.7 (19.1–22.9)	20.5 (19.3–22.8)	0.021
Nulliparous	2224 (50.2%)	117 (74.1%)	126 (60.6%)	397 (63.6%)	< 0.001
Uterine fibroids	182 (4.1%)	7 (4.4%)	25 (12.0%)	37 (5.9%)	< 0.001
Endometriosis	48 (1.1%)	6 (3.8%)	16 (7.7%)	24 (3.8%)	< 0.001
Adenomyosis	23 (0.5%)	1 (0.6%)	2 (1.0%)	3 (0.5%)	0.852
Endometriosis or adenomyosis	68 (1.5%)	7 (4.4%)	17 (8.2%)	27 (4.3%)	< 0.001

*Note:* Values are presented as median (interquartile range) or number (%). Continuous variables were compared among groups using the Kruskal–Wallis test, and categorical variables using the chi‐squared test. Patients with both endometriosis and adenomyosis were counted once in the “Endometriosis or adenomyosis” row.

Abbreviation: BMI, body mass index.

Comparison of ART treatment characteristics among the three ART groups showed significant differences in the blastocyst transfer rate (80.5% for fresh embryo transfer, 88.0% for OC‐FET, and 91.8% for HRC‐FET; *p* < 0.001) and the proportion of transfers involving two embryos (6.5% for fresh embryo transfer, 23.7% for OC‐FET, and 28.2% for HRC‐FET; *p* < 0.001). In contrast, no significant differences were observed in the ICSI rate (*p* = 0.233) or the good‐quality embryo transfer rate (*p* = 0.141) (Table S1).

### 3.2 Bleeding‐Related Outcomes

In the primary analysis, the HRC‐FET group showed significantly higher risks than the non‐ART group for manual removal of the placenta, PPH, blood loss ≥ 1500 mL, blood loss ≥ 2000 mL, and uterine atony. The incidence of each event by conception method is shown in Figure 1. Compact letter display analysis showed that, for manual removal of the placenta, PPH, and uterine atony, the HRC‐FET group differed significantly from all three other groups, which did not differ from one another; for blood loss ≥ 1500 mL and ≥ 2000 mL, the fresh embryo transfer and OC‐FET groups showed an intermediate pattern that did not differ significantly from either the non‐ART or HRC‐FET group. The incidence of manual removal of the placenta was 9.1% in the HRC‐FET group and 0.6% in the non‐ART group, with an aOR of 14.54 (95% CI, 7.73–27.33) on multivariable analysis. The incidence of PPH was 59.5% in the HRC‐FET group and 36.2% in the non‐ART group, with an aOR of 2.33 (95% CI, 1.88–2.89). The aOR was 2.56 (95% CI, 1.76–3.74) for blood loss ≥ 1500 mL, 2.29 (95% CI, 1.23–4.28) for blood loss ≥ 2000 mL, and 3.23 (95% CI, 2.44–4.27) for uterine atony (Table 2). Crude odds ratios from the univariable analysis are shown in Table S2. In the fresh embryo transfer group, no significant differences were observed for manual removal of the placenta, PPH, blood loss ≥ 1500 mL, blood loss ≥ 2000 mL, or uterine atony.

**FIGURE 1:**
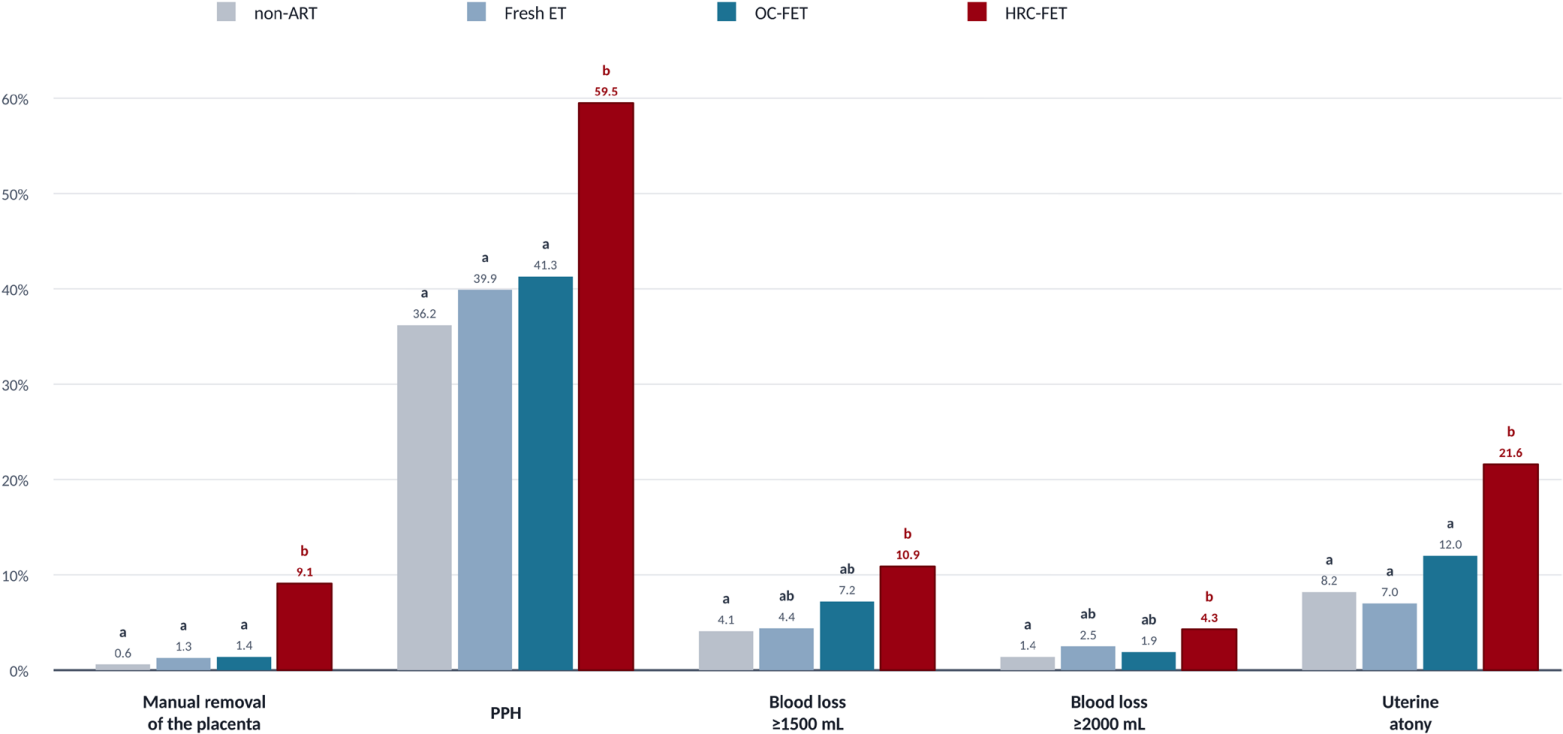
Incidence of bleeding‐related outcomes across the four conception groups. Grouped bar chart showing the incidence (%) of five bleeding‐related outcomes in the non‐ART, fresh embryo transfer, OC‐FET, and HRC‐FET groups. Bars represent the proportion of cases with each outcome within each group. Different letters above bars (a, b) indicate a statistically significant difference between groups in pairwise comparisons (Table S3). ART, assisted reproductive technology; HRC‐FET, hormone replacement cycle frozen embryo transfer; non‐ART, non–assisted reproductive technology; OC‐FET, ovulatory‐cycle frozen embryo transfer; PPH, postpartum hemorrhage.

**TABLE 2:** Incidence of bleeding‐related outcomes and adjusted odds ratios in the fresh embryo transfer, OC‐FET, and HRC‐FET groups, with the non‐ART group as reference.

Outcome	Non‐ART (*n* = 4427)	Fresh ET (*n* = 158)	OC‐FET (*n* = 208)	HRC‐FET (*n* = 624)	aOR, Fresh ET (vs. non‐ART)	aOR, OC‐FET (vs. non‐ART)	aOR, HRC‐FET (vs. non‐ART)
Manual removal of the placenta	0.6% (25/4427)^a^	1.3% (2/158)^a^	1.4% (3/208)^a^	9.1% (57/624)^b^	1.83 (0.24–14.24)	1.94 (0.43–8.68)	14.54 (7.73–27.33)**
PPH	36.2% (1601/4427)^a^	39.9% (63/158)^a^	41.3% (86/208)^a^	59.5% (371/624)^b^	1.44 (0.93–2.24)	1.00 (0.71–1.40)	2.33 (1.88–2.89)**
Blood loss ≥ 1500 mL	4.1% (183/4427)^a^	4.4% (7/158)^ab^	7.2% (15/208)^ab^	10.9% (68/624)^b^	1.45 (0.61–3.45)	1.48 (0.77–2.86)	2.56 (1.76–3.74)**
Blood loss ≥ 2000 mL	1.4% (62/4427)^a^	2.5% (4/158)^ab^	1.9% (4/208)^ab^	4.3% (27/624)^b^	2.80 (0.95–8.24)	0.37 (0.05–2.73)	2.29 (1.23–4.28)**
Uterine atony	8.2% (362/4427)^a^	7.0% (11/158)^a^	12.0% (25/208)^a^	21.6% (135/624)^b^	1.14 (0.54–2.42)	1.24 (0.72–2.14)	3.23 (2.44–4.27)**

*Note:* Values are presented as number (%). Different superscript letters (a, b) indicate a statistically significant difference between groups in pairwise comparisons (Table S3); groups sharing the same letter (or a common letter, e.g., “ab”) did not differ significantly. PPH was defined as blood loss ≥ 500 mL for vaginal delivery and ≥ 1000 mL for cesarean delivery. Adjusted odds ratios were derived from multivariable logistic regression adjusted for maternal age, pre‐pregnancy BMI, and parity, with the non‐ART group as reference. *p < 0.05, **p < 0.01.

Abbreviations: aOR, adjusted odds ratio; BMI, body mass index; HRC‐FET, hormone replacement cycle frozen embryo transfer; non‐ART, non–assisted reproductive technology; OC‐FET, ovulatory‐cycle frozen embryo transfer; PPH, postpartum hemorrhage.

In the OC‐FET group, likewise, no significant differences were observed for manual removal of the placenta, PPH, blood loss ≥ 1500 mL, blood loss ≥ 2000 mL, or uterine atony. In pairwise comparisons using the fresh embryo transfer and OC‐FET groups as reference, the HRC‐FET group also showed significantly elevated risks of manual removal of the placenta, PPH, and uterine atony (Table S3).

### 3.3 Perinatal Outcomes

Among the secondary outcomes, significant differences were observed in the HRC‐FET group for cesarean delivery, emergency cesarean delivery, meconium‐stained amniotic fluid, labor induction or augmentation, very low birth weight, low Apgar score, and low umbilical artery pH. The incidence of cesarean delivery was 40.7% in the HRC‐FET group and 31.9% in the non‐ART group, with an aOR of 1.42 (95% CI, 1.14–1.77). The aOR for emergency cesarean delivery was 1.64 (95% CI, 1.24–2.16). The incidence of meconium‐stained amniotic fluid was 9.0% in the HRC‐FET group and 4.8% in the non‐ART group, with an aOR of 1.85 (95% CI, 1.23–2.77). The incidence of labor induction or augmentation was 50.2% in the HRC‐FET group and 36.2% in the non‐ART group, with an aOR of 1.52 (95% CI, 1.22–1.91). The aOR for very low birth weight was 2.03 (95% CI, 1.04–3.98). Among neonatal outcomes, the aOR was 1.93 (95% CI, 1.11–3.35) for low Apgar score at 5 min, 1.77 (95% CI, 1.22–2.56) for low Apgar score at 1 min, and 3.10 (95% CI, 1.24–7.77) for low umbilical artery pH (Table 3).

**TABLE 3:** Incidence of major secondary outcomes and adjusted odds ratios in the fresh embryo transfer, OC‐FET, and HRC‐FET groups, with the non‐ART group as reference.

Outcome	Non‐ART (*n* = 4427)	Fresh ET (*n* = 158)	OC‐FET (*n* = 208)	HRC‐FET (*n* = 624)	aOR, Fresh ET (vs. non‐ART)	aOR, OC‐FET (vs. non‐ART)	aOR, HRC‐FET (vs. non‐ART)
Cesarean delivery	31.9% (1410/4427)	27.8% (44/158)	35.1% (73/208)	40.7% (254/624)	0.84 (0.52–1.35)	1.27 (0.90–1.78)	1.42 (1.14–1.77)**
Emergency cesarean delivery	13.7% (606/4427)	12.7% (20/158)	13.0% (27/208)	20.4% (127/624)	1.07 (0.59–1.95)	0.93 (0.57–1.52)	1.64 (1.24–2.16)**
Meconium‐stained amniotic fluid	4.8% (213/4427)	5.1% (8/158)	7.7% (16/208)	9.0% (56/624)	0.86 (0.30–2.42)	1.33 (0.67–2.66)	1.85 (1.23–2.77)**
Induction or augmentation of labor	36.2% (1604/4427)	36.1% (57/158)	43.8% (91/208)	50.2% (313/624)	0.78 (0.49–1.25)	0.96 (0.67–1.37)	1.52 (1.22–1.91)**
Very low birth weight (< 1500 g)	1.8% (80/4427)	3.2% (5/158)	1.4% (3/208)	2.9% (18/624)	1.54 (0.36–6.65)	0.81 (0.19–3.45)	2.03 (1.04–3.98)*
Low Apgar score at 5 min (< 7)	2.7% (106/3882)	3.2% (4/126)	2.0% (4/198)	4.6% (26/563)	1.41 (0.42–4.70)	0.77 (0.23–2.53)	1.93 (1.11–3.35)*
Low Apgar score at 1 min (< 7)	6.7% (259/3882)	5.6% (7/126)	6.1% (12/198)	9.4% (53/563)	0.90 (0.36–2.29)	0.91 (0.45–1.85)	1.77 (1.22–2.56)**
Low umbilical artery pH (< 7.1)	0.6% (25/4427)	1.3% (2/158)	0.0% (0/208)	1.6% (10/624)	1.93 (0.24–15.35)	—	3.10 (1.24–7.77)*

*Note:* Values are presented as number (%). Adjusted odds ratios were derived from multivariable logistic regression adjusted for maternal age, pre‐pregnancy BMI, and parity, with the non‐ART group as reference. A dash (—) indicates that the odds ratio could not be estimated because of zero events. *p < 0.05, **p < 0.01.

Abbreviations: aOR, adjusted odds ratio; BMI, body mass index; HRC‐FET, hormone replacement cycle frozen embryo transfer; non‐ART, non–assisted reproductive technology; OC‐FET, ovulatory‐cycle frozen embryo transfer.

No significant differences were observed among the groups for HDP, preeclampsia, superimposed preeclampsia, GH, GDM, placenta previa, placental abruption, PROM, oligohydramnios, preterm birth, preterm birth before 34 weeks, low birth weight, SGA, FGR, LGA, or NICU admission (Table S4).

In addition, pairwise comparisons using the fresh embryo transfer group as reference showed significantly elevated risks in the HRC‐FET group for cesarean delivery and labor induction or augmentation, along with significantly lower risks for placenta previa and placental abruption. Pairwise comparisons using the OC‐FET group as reference showed significantly elevated risks in the HRC‐FET group for emergency cesarean delivery, HDP, and labor induction or augmentation (Table S3).

### 3.4 Stratified Analysis

In the analysis restricted to vaginal deliveries, the HRC‐FET group showed significantly higher risks than the non‐ART group for manual removal of the placenta, PPH, blood loss ≥ 1500 mL, and uterine atony. The aORs were 15.95 (95% CI, 7.93–32.10) for manual removal of the placenta, 3.83 (95% CI, 2.85–5.14) for PPH, 8.66 (95% CI, 4.48–16.74) for blood loss ≥ 1500 mL, and 3.93 (95% CI, 2.85–5.43) for uterine atony. For blood loss ≥ 2000 mL, the number of events was too small to yield a stable adjusted odds ratio. In contrast, the fresh embryo transfer and OC‐FET groups showed no significant differences from the non‐ART group for any bleeding‐related outcome (Table S5).

In the analysis restricted to cesarean deliveries, the HRC‐FET group showed a significantly higher risk than the non‐ART group for uterine atony (aOR 2.27, 95% CI 1.24–4.16). No significant differences were observed for PPH, blood loss ≥ 1500 mL, or blood loss ≥ 2000 mL (Table S6). Forest plots of the primary outcomes, stratified by mode of delivery, are shown in Figure 2.

**FIGURE 2:**
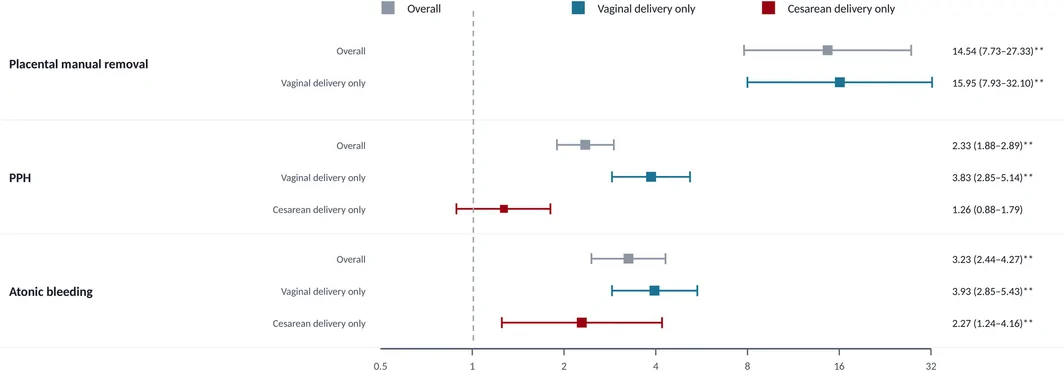
Adjusted odds ratios for bleeding‐related outcomes in HRC‐FET versus non‐ART, stratified by mode of delivery. Forest plot showing adjusted odds ratios with 95% confidence intervals for HRC‐FET compared with non‐ART across four bleeding‐related outcomes, presented for the overall cohort and for vaginal and cesarean deliveries analyzed separately. Odds ratios were adjusted for maternal age, pre‐pregnancy BMI, and parity. Squares represent point estimates, and horizontal lines represent 95% confidence intervals. **p < 0.01. ART, assisted reproductive technology; BMI, body mass index; HRC‐FET, hormone replacement cycle frozen embryo transfer; PPH, postpartum hemorrhage.

### 3.5 Sensitivity Analysis

In the sensitivity analysis with endometriosis or adenomyosis added to the model, the associations of HRC‐FET with the primary bleeding‐related outcomes were essentially unchanged (Table S7). For example, compared with the non‐ART group, the aOR of HRC‐FET was 14.66 (95% CI, 7.77–27.64) for manual removal of the placenta, 2.32 (1.86–2.87) for PPH, and 3.27 (2.47–4.33) for uterine atony. The results of the other pairwise comparisons were also consistent with the main analysis.

## 4 Discussion

By comparing four groups—non‐ART, fresh embryo transfer, OC‐FET, and HRC‐FET—within the same population, this study demonstrated that the increased perinatal risk associated with ART pregnancies is not uniform, but is concentrated primarily in bleeding‐related outcomes in the HRC‐FET group. In particular, the HRC‐FET group showed a consistent and significant increase in the risk of manual removal of the placenta, PPH, and uterine atony compared with the non‐ART group, whereas the OC‐FET and fresh embryo transfer groups did not show a similarly consistent increase. These findings suggest that, rather than regarding all ART pregnancies as uniformly high‐risk, it is important to consider risk differences by treatment subgroup.

Previous studies, comparing HRC with natural ovulatory cycles or comparing fresh embryo transfer with FET, have reported an increased risk of maternal complications in HRC‐FET [4, 6, 10]. However, most of these studies were limited to a single pairwise comparison, and few have simultaneously compared non‐ART, fresh embryo transfer, OC‐FET, and HRC‐FET within a unified analytic framework to characterize the relative position of each group. The present design allowed the relative position of each group to be clarified more directly than in prior pairwise comparisons. When the magnitude of the effect sizes observed in this study is compared with previous reports, the aOR for manual removal of the placenta (14.54 overall; 15.95 in the analysis restricted to vaginal delivery) is close to the adjusted odds ratio of 11.4 (95% CI, 8.7–15.0) reported for PAS in HRC‐FET in a large Japanese nationwide ART registry study (224,043 vaginal deliveries), placing our estimate near the upper end of previously reported values [16]. However, it was somewhat higher than the adjusted odds ratios of 4.14–6.91 reported in single‐center studies [10, 17]. This difference may reflect a lower clinical threshold for “manual removal of the placenta” in the present study compared with diagnostic criteria for PAS based on imaging or pathological findings, or it may reflect differences in clinical practice and documentation patterns across institutions. For PPH, the aOR in this study (2.33) falls within the range reported in previous meta‐analyses and cohort studies (approximately 2.3–2.9) and is consistent with previous findings [18, 19]. For cesarean delivery, the aOR in this study (1.42) showed a direction generally consistent with that of a previous large‐scale study (adjusted odds ratio, 1.64) [10].

A possible mechanistic explanation for this finding is the absence of corpus luteum formation during an HRC. Because ovulation—and consequently corpus luteum formation—does not occur in HRC‐FET, corpus luteum–derived vasoactive substances and the early pregnancy endocrine milieu differ from those in OC‐FET. These differences may affect placentation, placental attachment, and myometrial contractility and hemostasis at delivery, potentially resulting in an increased risk of manual removal of the placenta, PPH, and uterine atony [8, 9]. Although this study did not directly examine the underlying mechanism, the consistent increase in bleeding‐related outcomes observed in HRC‐FET is compatible with this pathophysiological hypothesis. We further performed a stratified analysis by mode of delivery, separating vaginal and cesarean deliveries. In the analysis restricted to vaginal delivery, the HRC‐FET group continued to show a significantly elevated risk of manual removal of the placenta, PPH, and uterine atony. This indicates that the increased bleeding‐related risk in the HRC‐FET group is not explained solely by the higher cesarean delivery rate in this group. In contrast, in the analysis restricted to cesarean delivery, uterine atony remained significantly elevated, whereas no significant differences were observed for PPH or blood loss ≥ 2000 mL. These findings suggest that the pattern of bleeding‐related risk associated with HRC‐FET may differ according to the mode of delivery, potentially reflecting differences in the mechanisms and clinical management of bleeding between vaginal and cesarean deliveries.

Among the secondary outcomes, the HRC‐FET group also showed significant differences compared with the non‐ART group in cesarean delivery, emergency cesarean delivery, meconium‐stained amniotic fluid, induction or augmentation of labor, very low birth weight, low Apgar score, and low umbilical artery pH. However, these findings were less consistent than the bleeding‐related outcomes, and no clear increase was observed for outcomes such as HDP, preeclampsia, preterm birth, SGA, and FGR. The principal message of this study, therefore, is that HRC‐FET does not uniformly worsen perinatal outcomes overall, but instead shows a characteristic effect that is concentrated primarily in maternal bleeding‐related outcomes.

Previous studies have reported a higher risk of HDP, including preeclampsia, after HRC‐FET than after natural or ovulatory‐cycle FET [10, 18, 19, 20, 21], and a higher risk after FET in general than after spontaneous conception [22]. In contrast, we did not find a significant increase in HDP in the HRC‐FET group compared with the non‐ART group (aOR, 1.09; 95% CI, 0.73–1.62). This difference from the previous reports should be interpreted with caution, and our results should not be regarded as evidence of a true absence of association. First, most of the previous studies compared HRC‐FET with natural or ovulatory‐cycle FET, whereas the reference group in our primary analysis was the non‐ART group. In our cohort, the incidence of HDP in the HRC‐FET group (8.0%) was similar to that in the non‐ART group (7.3%) and the fresh embryo transfer group (8.9%), whereas it was lower in the OC‐FET group (4.3%). Accordingly, in the direct comparison between HRC‐FET and OC‐FET, HRC‐FET showed a higher risk of HDP (aOR, 2.71; 95% CI, 1.04–7.11), which is in the same direction as the previous reports. Whether the lower incidence in OC‐FET reflects the presence of the corpus luteum [8, 9] cannot be determined from our data, because the number of events in that group was small. Second, this was a single‐center study, and the number of HDP events was small, especially in the OC‐FET group (9 events; 2 of preeclampsia), so that the estimates were imprecise. The 95% CI for HRC‐FET versus non‐ART does not exclude a modest increase in risk, and this study was not designed to detect differences in HDP. Third, our institution is a regional perinatal medical center, and even the non‐ART group may include many pregnancies with some high‐risk condition, which may have led to a relatively high incidence of HDP in the non‐ART group (7.3%). Fourth, OC‐FET in this study included both natural cycles and letrozole‐induced cycles, and the details of the HRC regimens, such as the route and duration of estrogen and progesterone administration, were not examined, whereas previous studies have used different protocol definitions. Finally, we adjusted only for maternal age, pre‐pregnancy BMI, and parity, so that residual confounding by other risk factors for HDP, such as chronic hypertension and the cause of infertility, cannot be excluded. Larger studies with detailed protocol information and adequate control of confounding are needed to clarify whether the risk of HDP differs according to the endometrial preparation protocol.

Clinically, this study enables HRC‐FET to be identified as a treatment subgroup with a distinct risk profile. In this study, at least, OC‐FET and fresh embryo transfer did not show as consistent an increase in maternal bleeding‐related risk as HRC‐FET and showed a risk profile resembling that of the non‐ART group for most outcomes. These findings may contribute to more refined risk stratification among ART pregnancies and inform perinatal management according to the treatment protocol used. In particular, perinatal management after HRC‐FET should take into account the possibility of bleeding‐related complications, including manual removal of the placenta and postpartum hemorrhage.

Nevertheless, HRC‐FET is widely used in contemporary ART practice, particularly in Japan, where it accounts for a substantial proportion of FET cycles [1]. HRC‐FET offers important practical advantages, including greater flexibility in scheduling embryo transfer and the ability to perform FET without relying on spontaneous ovulation, making it a practical and necessary option for patients in whom ovulatory‐cycle FET is difficult or unsuitable [23, 24]. Therefore, the present findings should not be interpreted as indicating that HRC‐FET should be avoided. Rather, they highlight the importance of recognizing its distinct perinatal risk profile while preserving its clinical advantages and developing strategies to mitigate the associated risks. In particular, if the absence of the corpus luteum contributes to the increased risk of bleeding‐related complications, future studies should investigate whether the physiological consequences of corpus luteum absence can be mitigated and whether such approaches can reduce adverse maternal outcomes. Until effective preventive strategies are established, recognition of the increased bleeding‐related risk associated with HRC‐FET may contribute to more appropriate antenatal risk assessment and preparedness for hemorrhagic complications at delivery.

This study has several limitations. First, it was a single‐center retrospective study, and residual confounding cannot be completely excluded. Second, the non‐ART group likely included many naturally conceived pregnancies without infertility, whereas the ART groups comprised women who had required infertility treatment. Therefore, these two groups may represent fundamentally different populations in terms of fertility and underlying medical conditions. Confounding factors not adjusted for in this study—such as the cause and duration of infertility and other underlying conditions—beyond age, BMI, and parity may have contributed to the observed between‐group differences, and the observed differences should not be attributed solely to differences in endometrial preparation protocol [25]. Third, the choice of endometrial preparation protocol itself reflects clinical decision‐making based on patient characteristics such as anovulation or menstrual irregularity, and this indication‐based selection bias may not have been fully accounted for in this study. Fourth, no standardized institutional protocol governed the timing and indication of manual removal of the placenta. Although approximately 30 min was used as a general guide, the final decision was left to the attending obstetrician, so the incidence of manual removal may have been influenced by inter‐physician differences in clinical practice. Fifth, a history of endometriosis or adenomyosis was ascertained from patient self‐report, referral letters, and findings at our institution, without a uniform diagnostic criterion. In patients who received ART at other clinics, endometriosis may not have been recorded, and adenomyosis is difficult to diagnose accurately after conception. Misclassification is therefore possible and the prevalence of these conditions may be underestimated. However, adding these conditions to the model did not materially change the associations between HRC‐FET and bleeding‐related outcomes. Sixth, uterine atony was diagnosed clinically by the attending obstetrician on the basis of the response to the routine oxytocin regimen and the need for additional treatment, without an objective criterion such as a blood loss threshold. The diagnosis and the decision to initiate additional treatment may therefore have varied among obstetricians. Seventh, although manual removal of the placenta is a clinical finding suggestive of placental adherence, it does not constitute a pathologically confirmed diagnosis of PAS [26]. Eighth, factors that may affect placenta‐related abnormalities, such as a history of cesarean delivery or certain uterine surgeries, may not have been adequately assessed. Ninth, FGR in this study was defined as an EFW ≤ −1.5 SD on at least one occasion during pregnancy, based on ultrasound biometry. This cross‐sectional definition does not capture the decline in longitudinal growth trajectory, which is increasingly emphasized in the contemporary pathophysiological concept of FGR [27]. Future prospective studies that minimize selection bias in the choice of endometrial preparation protocol, as well as mechanistic studies directly measuring corpus luteum–derived vasoactive substances, are warranted to validate these findings.

In summary, the increased maternal risk associated with ART pregnancies was not uniform across all treatment subgroups, but was concentrated primarily in HRC‐FET. Compared with non‐ART, fresh embryo transfer, and OC‐FET, HRC‐FET was associated with an increased risk of manual removal of the placenta, PPH, and uterine atony. Perinatal management after HRC‐FET should therefore take bleeding‐related complications into consideration.

## Ethics Statement

This study was approved by the Ethics Committee of the University of Yamanashi (approval no. 3095).

## Consent

All procedures followed were in accordance with the ethical standards of the responsible committee on human experimentation (institutional and national) and with the Helsinki declaration of 1964 and later versions. The requirement for written informed consent was waived because of the retrospective nature of the study, and an opt‐out approach was used in accordance with the ethics committee approval.

## Conflicts of Interest

The authors declare no conflicts of interest.

## Supporting information


**Table S1:** Comparison of ART treatment characteristics among the fresh embryo transfer, OC‐FET, and HRC‐FET groups.
**Table S2:** Incidence of all outcomes and crude odds ratios in the fresh embryo transfer, OC‐FET, and HRC‐FET groups, with the non‐ART group as reference.
**Table S3:** Adjusted odds ratios for all pairwise comparisons among the non‐ART, fresh embryo transfer, OC‐FET, and HRC‐FET groups.
**Table S4:** Incidence of secondary outcomes and adjusted odds ratios in the fresh embryo transfer, OC‐FET, and HRC‐FET groups, with the non‐ART group as reference.
**Table S5:** Incidence of bleeding‐related outcomes and adjusted odds ratios in the fresh embryo transfer, OC‐FET, and HRC‐FET groups, restricted to vaginal deliveries, with the non‐ART group as reference (*n* = 3631).
**Table S6:** Incidence of bleeding‐related outcomes and adjusted odds ratios in the fresh embryo transfer, OC‐FET, and HRC‐FET groups, restricted to cesarean deliveries, with the non‐ART group as reference (*n* = 1786).
**Table S7:** Sensitivity analysis of bleeding‐related outcomes with additional adjustment for endometriosis or adenomyosis.

## Data Availability

The data that support the findings of this study are available from the corresponding author upon reasonable request.
