# Supplementary material for: Bleeding‐Related Perinatal Outcomes Following Hormone Replacement Cycle Frozen Embryo Transfer Compared With Non‐ART, Fresh Embryo Transfer, and Ovulatory‐Cycle Frozen Embryo Transfer: A Single‐Center Cohort Study
Source: Reprod Med Biol. 2026 Oct 29;25(1):e70110. doi: 10.1002/rmb2.70110 (PMC13719801; doi:10.1002/rmb2.70110)
Supplement: Supplementary file 1 [file RMB2-25-e70110-s001.docx]

Supplementary Table 1. Comparison of ART treatment characteristics among the fresh embryo transfer, OC-FET, and HRC-FET groups

Values are presented as number of cases/number of evaluable cases (%). Groups were compared using the chi-square test. A good-quality embryo was defined as a blastocyst in which neither the inner cell mass grade nor the trophectoderm grade was C; when two embryos were transferred, the transfer was classified as good-quality if at least one embryo met this criterion. The good-quality embryo transfer rate was calculated only among blastocyst transfers. Cases with missing data were excluded from the denominator. HRC-FET, hormone replacement cycle frozen embryo transfer; ICSI, intracytoplasmic sperm injection; OC-FET, ovulatory-cycle frozen embryo transfer.

|  | Fresh ET (n=158) | OC-FET (n=208) | HRC-FET (n=624) | p value |
| --- | --- | --- | --- | --- |
| ICSI rate | 63/156 (40.4%) | 94/198 (47.5%) | 231/566 (40.8%) | 0.233 |
| Blastocyst transfer rate | 128/158 (80.5%) | 183/208 (88.0%) | 573/624 (91.8%) | <0.001 |
| Two-embryo transfer | 10/154 (6.5%) | 47/198 (23.7%) | 155/549 (28.2%) | <0.001 |
| Good-quality embryo transfer rate (blastocyst transfers only) | 45/46 (97.8%) | 101/103 (98.1%) | 348/371 (93.8%) | 0.141 |

Supplementary Table 2. Incidence of all outcomes and crude odds ratios in the fresh embryo transfer, OC-FET, and HRC-FET groups, with the non-ART group as reference

Values are presented as number (%). Groups were compared using the chi-square test for all outcomes except continuous variables; crude (unadjusted) odds ratios were calculated using univariable logistic regression, with the non-ART group as reference. *p<0.05, **p<0.01.

FGR, fetal growth restriction; GDM, gestational diabetes mellitus; GH, gestational hypertension; HDP, hypertensive disorders of pregnancy; HRC-FET, hormone replacement cycle frozen embryo transfer; LGA, large for gestational age; NICU, neonatal intensive care unit; non-ART, non–assisted reproductive technology; OC-FET, ovulatory-cycle frozen embryo transfer; OR, odds ratio; PE, preeclampsia; PPH, postpartum hemorrhage; PROM, premature rupture of membranes; SGA, small for gestational age.

| Outcome | non-ART (n=4,427) | Fresh ET (n=158) | OC-FET (n=208) | HRC-FET (n=624) | p value | Crude OR, Fresh ET (vs non-ART) | Crude OR, OC-FET (vs non-ART) | Crude OR, HRC-FET (vs non-ART) |
| --- | --- | --- | --- | --- | --- | --- | --- | --- |
| HDP | 7.3% (325/4427) | 8.9% (14/158) | 4.3% (9/208) | 8.0% (51/624) | 0.293 | 1.23 (0.70–2.15) | 0.57 (0.29–1.12) | 1.10 (0.81–1.50) |
| Preeclampsia | 2.6% (114/4427) | 2.5% (4/158) | 1.0% (2/208) | 3.4% (21/624) | 0.295 | 0.99 (0.36–2.72) | 0.37 (0.09–1.51) | 1.33 (0.83–2.13) |
| Superimposed PE | 0.3% (13/4427) | 0.0% (0/158) | 0.5% (1/208) | 0.3% (2/624) | 0.867 | — | 1.64 (0.21–12.60) | 1.09 (0.25–4.85) |
| GH | 4.4% (198/4427) | 5.7% (10/158) | 2.9% (6/208) | 4.5% (28/624) | 0.619 | 1.30 (0.65–2.58) | 0.64 (0.28–1.45) | 1.01 (0.67–1.51) |
| GDM | 8.9% (395/4427) | 10.1% (16/158) | 9.6% (20/208) | 8.2% (51/624) | 0.841 | 1.15 (0.68–1.95) | 1.09 (0.68–1.74) | 0.91 (0.67–1.23) |
| Placenta previa | 2.2% (98/4427) | 3.2% (5/158) | 1.9% (4/208) | 2.2% (14/624) | 0.867 | 1.44 (0.58–3.60) | 0.87 (0.32–2.38) | 1.01 (0.58–1.79) |
| Placental abruption | 1.3% (59/4427) | 1.9% (3/158) | 1.0% (2/208) | 1.0% (6/624) | 0.747 | 1.43 (0.44–4.62) | 0.72 (0.17–2.96) | 0.72 (0.31–1.67) |
| Manual removal of the placenta | 0.6% (25/4427) | 1.3% (2/158) | 1.4% (3/208) | 9.1% (57/624) | <0.001 | 2.26 (0.53–9.62) | 2.58 (0.77–8.60) | 17.70 (10.97–28.56)** |
| PPH | 36.2% (1601/4427) | 39.9% (63/158) | 41.3% (86/208) | 59.5% (371/624) | <0.001 | 1.17 (0.85–1.62) | 1.24 (0.94–1.65) | 2.59 (2.18–3.07)** |
| Blood loss ≥1,500 mL | 4.1% (183/4427) | 4.4% (7/158) | 7.2% (15/208) | 10.9% (68/624) | <0.001 | 1.08 (0.50–2.33) | 1.80 (1.04–3.11)* | 2.84 (2.12–3.80)** |
| Blood loss ≥2,000 mL | 1.4% (62/4427) | 2.5% (4/158) | 1.9% (4/208) | 4.3% (27/624) | <0.001 | 1.83 (0.66–5.09) | 1.38 (0.50–3.83) | 3.18 (2.01–5.04)** |
| Uterine atony | 8.2% (362/4427) | 7.0% (11/158) | 12.0% (25/208) | 21.6% (135/624) | <0.001 | 0.84 (0.45–1.57) | 1.53 (1.00–2.36) | 3.10 (2.49–3.86)** |
| Cesarean delivery | 31.9% (1410/4427) | 27.8% (44/158) | 35.1% (73/208) | 40.7% (254/624) | <0.001 | 0.83 (0.58–1.18) | 1.16 (0.86–1.55) | 1.47 (1.24–1.74)** |
| Elective cesarean delivery | 18.2% (804/4427) | 15.2% (24/158) | 22.1% (46/208) | 20.4% (127/624) | 0.194 | 0.81 (0.52–1.25) | 1.28 (0.91–1.79) | 1.15 (0.93–1.42) |
| Emergency cesarean delivery | 13.7% (606/4427) | 12.7% (20/158) | 13.0% (27/208) | 20.4% (127/624) | <0.001 | 0.91 (0.57–1.47) | 0.94 (0.62–1.42) | 1.61 (1.30–1.99)** |
| PROM | 15.5% (687/4427) | 19.6% (31/158) | 11.1% (23/208) | 17.0% (106/624) | 0.106 | 1.33 (0.89–1.98) | 0.68 (0.44–1.05) | 1.11 (0.89–1.39) |
| Meconium-stained amniotic fluid | 4.8% (213/4427) | 5.1% (8/158) | 7.7% (16/208) | 9.0% (56/624) | <0.001 | 1.06 (0.51–2.18) | 1.65 (0.97–2.80) | 1.95 (1.43–2.65)** |
| Oligohydramnios | 1.5% (65/4427) | 0.0% (0/158) | 3.4% (7/208) | 2.9% (18/624) | 0.004 | — | 2.34 (1.06–5.16)* | 1.99 (1.17–3.38)* |
| Induction or augmentation of labor | 36.2% (1604/4427) | 36.1% (57/158) | 43.8% (91/208) | 50.2% (313/624) | <0.001 | 0.99 (0.71–1.38) | 1.37 (1.03–1.81)* | 1.77 (1.50–2.10)** |
| Preterm birth before 37 weeks of gestation | 9.0% (400/4423) | 13.8% (22/158) | 7.7% (16/208) | 7.5% (47/624) | 0.084 | 1.62 (1.02–2.56)* | 0.84 (0.50–1.41) | 0.82 (0.60–1.12) |
| Preterm birth before 34 weeks of gestation | 3.0% (131/4423) | 5.7% (9/159) | 3.4% (7/208) | 3.4% (21/624) | 0.268 | 1.97 (0.98–3.94) | 1.14 (0.53–2.47) | 1.14 (0.71–1.82) |
| SGA | 5.8% (256/4427) | 7.0% (11/158) | 4.8% (10/208) | 4.6% (29/624) | 0.550 | 1.22 (0.65–2.28) | 0.82 (0.43–1.57) | 0.79 (0.54–1.18) |
| LGA | 8.1% (357/4427) | 4.4% (7/158) | 7.2% (15/208) | 11.9% (74/624) | 0.003 | 0.53 (0.25–1.14) | 0.89 (0.52–1.51) | 1.53 (1.18–2.00)** |
| FGR | 3.3% (145/4427) | 4.4% (7/158) | 4.8% (10/208) | 2.6% (16/624) | 0.363 | 1.37 (0.63–2.97) | 1.49 (0.77–2.88) | 0.78 (0.46–1.31) |
| Low birth weight | 13.1% (582/4427) | 19.6% (31/158) | 13.5% (28/208) | 11.4% (71/624) | 0.057 | 1.61 (1.08–2.41)* | 1.03 (0.68–1.54) | 0.85 (0.65–1.10) |
| Very low birth weight | 1.8% (80/4427) | 3.2% (5/158) | 1.4% (3/208) | 2.9% (18/624) | 0.187 | 1.78 (0.71–4.45) | 0.80 (0.25–2.54) | 1.61 (0.96–2.71) |
| NICU admission | 13.4% (372/2782) | 19.3% (16/83) | 12.1% (23/190) | 13.3% (59/442) | 0.434 | 1.55 (0.89–2.70) | 0.89 (0.57–1.40) | 1.00 (0.74–1.34) |
| Low Apgar score at 5 minutes (<7) | 2.7% (106/3882) | 3.2% (4/126) | 2.0% (4/198) | 4.6% (26/563) | 0.079 | 1.17 (0.42–3.22) | 0.73 (0.27–2.01) | 1.72 (1.11–2.67)* |
| Low Apgar score at 1 minute (<7) | 6.7% (259/3882) | 5.6% (7/126) | 6.1% (12/198) | 9.4% (53/563) | 0.095 | 0.82 (0.38–1.78) | 0.90 (0.50–1.64) | 1.45 (1.07–1.98)* |
| Low umbilical artery pH (<7.1) | 0.6% (25/4427) | 1.3% (2/158) | 0.0% (0/208) | 1.6% (10/624) | 0.012 | 2.26 (0.53–9.62) | — | 2.87 (1.37–6.00)** |

Supplementary Table 3. Adjusted odds ratios for all pairwise comparisons among the non-ART, fresh embryo transfer, OC-FET, and HRC-FET groups

Values are presented as adjusted odds ratios (95% confidence intervals), derived from multivariable logistic regression adjusted for maternal age, pre-pregnancy BMI, and parity. *p<0.05, **p<0.01.

BMI, body mass index; FGR, fetal growth restriction; GDM, gestational diabetes mellitus; GH, gestational hypertension; HDP, hypertensive disorders of pregnancy; HRC-FET, hormone replacement cycle frozen embryo transfer; LGA, large for gestational age; NICU, neonatal intensive care unit; non-ART, non–assisted reproductive technology; OC-FET, ovulatory-cycle frozen embryo transfer; PE, preeclampsia; PPH, postpartum hemorrhage; PROM, premature rupture of membranes; SGA, small for gestational age.

| Outcome | HRC-FET (vs non-ART) | HRC-FET (vs Fresh ET) | HRC-FET (vs OC-FET) | OC-FET (vs non-ART) | OC-FET (vs Fresh ET) | Fresh ET (vs non-ART) |
| --- | --- | --- | --- | --- | --- | --- |
| Manual removal of the placenta | 14.54 (7.73–27.33)** | 7.92 (1.07–58.64)* | 7.49 (1.78–31.47)** | 1.94 (0.43–8.68) | 1.06 (0.09–11.85) | 1.83 (0.24–14.24) |
| PPH | 2.33 (1.88–2.89)** | 1.61 (1.01–2.58)* | 2.34 (1.60–3.41)** | 1.00 (0.71–1.40) | 0.69 (0.40–1.18) | 1.44 (0.93–2.24) |
| Blood loss ≥1,500 mL | 2.56 (1.76–3.74)** | 1.76 (0.73–4.28) | 1.73 (0.87–3.44) | 1.48 (0.77–2.86) | 1.02 (0.36–2.87) | 1.45 (0.61–3.45) |
| Blood loss ≥2,000 mL | 2.29 (1.23–4.28)** | 0.82 (0.27–2.52) | 6.24 (0.82–47.52) | 0.37 (0.05–2.73) | 0.13 (0.01–1.20) | 2.80 (0.95–8.24) |
| Uterine atony | 3.23 (2.44–4.27)** | 2.83 (1.32–6.06)** | 2.60 (1.48–4.58)** | 1.24 (0.72–2.14) | 1.09 (0.44–2.65) | 1.14 (0.54–2.42) |
| Cesarean delivery | 1.42 (1.14–1.77)** | 1.69 (1.02–2.81)* | 1.12 (0.77–1.63) | 1.27 (0.90–1.78) | 1.51 (0.86–2.67) | 0.84 (0.52–1.35) |
| Emergency cesarean delivery | 1.64 (1.24–2.16)** | 1.53 (0.82–2.87) | 1.76 (1.04–2.98)* | 0.93 (0.57–1.52) | 0.87 (0.41–1.83) | 1.07 (0.59–1.95) |
| Elective cesarean delivery | 1.10 (0.83–1.44) | 1.67 (0.86–3.26) | 0.74 (0.48–1.15) | 1.55 (1.04–2.29)* | 2.29 (1.09–4.83)* | 0.73 (0.38–1.39) |
| HDP | 1.09 (0.73–1.62) | 0.82 (0.37–1.79) | 2.71 (1.04–7.11)* | 0.40 (0.16–1.01) | 0.30 (0.10–0.94)* | 1.33 (0.64–2.77) |
| Preeclampsia | 1.43 (0.76–2.69) | 0.74 (0.24–2.31) | — | 0.00 (0.00–inf) | 0.00 (0.00–inf) | 1.95 (0.67–5.65) |
| Superimposed PE | 1.49 (0.27–8.39) | — | — | — | — | — |
| GH | 0.96 (0.58–1.60) | 0.88 (0.32–2.43) | 1.95 (0.65–5.87) | 0.55 (0.20–1.54) | 0.45 (0.12–1.77) | 1.19 (0.46–3.06) |
| GDM | 0.71 (0.48–1.03) | 0.58 (0.29–1.17) | 0.83 (0.44–1.55) | 0.86 (0.49–1.48) | 0.70 (0.31–1.57) | 1.22 (0.65–2.31) |
| Placenta previa | 0.63 (0.29–1.35) | 0.31 (0.10–0.99)* | 0.76 (0.22–2.56) | 0.83 (0.29–2.35) | 0.40 (0.10–1.56) | 2.06 (0.79–5.39) |
| Placental abruption | 0.70 (0.24–2.05) | 0.20 (0.04–0.95)* | 0.66 (0.12–3.74) | 0.90 (0.21–3.89) | 0.35 (0.06–2.18) | 2.38 (0.69–8.19) |
| PROM | 0.96 (0.72–1.30) | 0.78 (0.42–1.43) | 1.45 (0.82–2.57) | 0.59 (0.35–1.01) | 0.58 (0.27–1.23) | 1.09 (0.62–1.92) |
| Meconium-stained amniotic fluid | 1.85 (1.23–2.77)** | 2.07 (0.71–6.00) | 1.35 (0.65–2.79) | 1.27 (0.63–2.54) | 1.67 (0.49–5.68) | 0.80 (0.28–2.27) |
| Oligohydramnios | 1.77 (0.91–3.45) | — | 1.52 (0.42–5.46) | 1.16 (0.34–3.90) | — | — |
| Induction or augmentation of labor | 1.52 (1.21–1.90)** | 1.84 (1.13–3.01)* | 1.56 (1.06–2.29)* | 0.94 (0.66–1.35) | 1.18 (0.67–2.07) | 0.74 (0.46–1.19) |
| NICU admission | 0.97 (0.67–1.40) | 0.57 (0.26–1.23) | 1.09 (0.59–2.01) | 0.92 (0.54–1.57) | 0.49 (0.21–1.18) | 1.77 (0.86–3.64) |
| Preterm birth before 37 weeks of gestation | 0.92 (0.62–1.36) | 0.63 (0.30–1.34) | 1.03 (0.52–2.05) | 0.87 (0.47–1.61) | 0.64 (0.26–1.56) | 1.43 (0.72–2.84) |
| Preterm birth before 34 weeks of gestation | 1.47 (0.81–2.68) | 0.75 (0.24–2.33) | 1.14 (0.41–3.19) | 1.25 (0.48–3.22) | 0.69 (0.18–2.68) | 1.86 (0.64–5.39) |
| Low birth weight | 0.84 (0.60–1.17) | 0.71 (0.37–1.38) | 0.93 (0.53–1.64) | 0.90 (0.55–1.49) | 0.76 (0.36–1.63) | 1.18 (0.64–2.18) |
| Very low birth weight | 2.10 (1.07–4.13)* | 1.31 (0.29–5.97) | 2.47 (0.55–11.08) | 0.81 (0.19–3.45) | 0.58 (0.08–4.25) | 1.48 (0.34–6.44) |
| SGA | 0.73 (0.45–1.20) | 0.54 (0.22–1.32) | 0.92 (0.40–2.13) | 0.80 (0.38–1.68) | 0.59 (0.21–1.68) | 1.36 (0.61–3.04) |
| LGA | 1.39 (0.98–1.97) | 2.57 (0.90–7.35) | 1.62 (0.82–3.23) | 0.86 (0.45–1.62) | 1.58 (0.49–5.14) | 0.54 (0.19–1.50) |
| FGR | 0.91 (0.47–1.76) | 0.71 (0.19–2.62) | 0.79 (0.27–2.32) | 1.15 (0.45–2.93) | 0.90 (0.21–3.87) | 1.27 (0.39–4.19) |
| Low Apgar score at 5 minutes (<7) | 1.93 (1.11–3.35)* | 1.32 (0.38–4.59) | 2.42 (0.71–8.31) | 0.77 (0.23–2.53) | 0.57 (0.11–2.91) | 1.41 (0.42–4.70) |
| Low Apgar score at 1 minute (<7) | 1.77 (1.22–2.56)** | 1.95 (0.75–5.08) | 1.87 (0.89–3.95) | 0.91 (0.45–1.85) | 1.07 (0.34–3.38) | 0.90 (0.36–2.29) |
| Low umbilical artery pH (<7.1) | 3.21 (1.28–8.07)* | 1.67 (0.21–13.65) | — | — | — | 1.84 (0.23–14.78) |

Supplementary Table 4. Incidence of secondary outcomes and adjusted odds ratios in the fresh embryo transfer, OC-FET, and HRC-FET groups, with the non-ART group as reference

Values are presented as number (%). Adjusted odds ratios were derived from multivariable logistic regression adjusted for maternal age, pre-pregnancy BMI, and parity, with the non-ART group as reference. A low Apgar score was defined as <7 points, and a low umbilical artery pH as <7.1. FGR was defined as Estimated fetal weight ≤−1.5 SD, and SGA as a birth weight below the 10th percentile for gestational age, sex, and birth order. A dash (—) indicates that the odds ratio could not be estimated because of zero events. *p<0.05, **p<0.01.

BMI, body mass index; FGR, fetal growth restriction; GDM, gestational diabetes mellitus; GH, gestational hypertension; HDP, hypertensive disorders of pregnancy; HRC-FET, hormone replacement cycle frozen embryo transfer; LGA, large for gestational age; NICU, neonatal intensive care unit; non-ART, non–assisted reproductive technology; OC-FET, ovulatory-cycle frozen embryo transfer; PE, preeclampsia; PPH, postpartum hemorrhage; PROM, premature rupture of membranes; SD, standard deviation; SGA, small for gestational age.

| Outcome | non-ART (n=4,427) | Fresh ET (n=158) | OC-FET (n=208) | HRC-FET (n=624) | aOR, Fresh ET (vs non-ART) | aOR, OC-FET (vs non-ART) | aOR, HRC-FET (vs non-ART) |
| --- | --- | --- | --- | --- | --- | --- | --- |
| Cesarean delivery | 31.9% (1410/4427) | 27.8% (44/158) | 35.1% (73/208) | 40.7% (254/624) | 0.84 (0.52–1.35) | 1.27 (0.90–1.78) | 1.42 (1.14–1.77)** |
| Emergency cesarean delivery | 13.7% (606/4427) | 12.7% (20/158) | 13.0% (27/208) | 20.4% (127/624) | 1.07 (0.59–1.95) | 0.93 (0.57–1.52) | 1.64 (1.24–2.16)** |
| Elective cesarean delivery | 18.2% (804/4427) | 15.2% (24/158) | 22.1% (46/208) | 20.4% (127/624) | 0.71 (0.37–1.35) | 1.48 (1.00–2.19)* | 1.09 (0.83–1.43) |
| HDP | 7.3% (325/4427) | 8.9% (14/158) | 4.3% (9/208) | 8.0% (51/624) | 1.33 (0.64–2.77) | 0.40 (0.16–1.01) | 1.09 (0.73–1.62) |
| Preeclampsia | 2.6% (114/4427) | 2.5% (4/158) | 1.0% (2/208) | 3.4% (21/624) | 1.95 (0.67–5.65) | 0.00 (0.00–inf) | 1.43 (0.76–2.69) |
| Superimposed PE | 0.3% (13/4427) | 0.0% (0/158) | 0.5% (1/208) | 0.3% (2/624) | 0.00 (0.00–inf) | 0.00 (0.00–inf) | 1.49 (0.27–8.39) |
| GH | 4.4% (198/4427) | 5.7% (10/158) | 2.9% (6/208) | 4.5% (28/624) | 1.13 (0.44–2.91) | 0.52 (0.19–1.47) | 0.96 (0.58–1.60) |
| GDM | 8.9% (395/4427) | 10.1% (16/158) | 9.6% (20/208) | 8.2% (51/624) | 1.22 (0.65–2.31) | 0.86 (0.49–1.48) | 0.71 (0.48–1.03) |
| Placenta previa | 2.2% (98/4427) | 3.2% (5/158) | 1.9% (4/208) | 2.2% (14/624) | 2.04 (0.78–5.34) | 0.85 (0.30–2.40) | 0.64 (0.30–1.38) |
| Placental abruption | 1.3% (59/4427) | 1.9% (3/158) | 1.0% (2/208) | 1.0% (6/624) | 2.68 (0.78–9.20) | 0.95 (0.22–4.09) | 0.69 (0.24–2.01) |
| PROM | 15.5% (687/4427) | 19.6% (31/158) | 11.1% (23/208) | 17.0% (106/624) | 1.05 (0.59–1.88) | 0.63 (0.37–1.07) | 0.96 (0.71–1.29) |
| Meconium-stained amniotic fluid | 4.8% (213/4427) | 5.1% (8/158) | 7.7% (16/208) | 9.0% (56/624) | 0.86 (0.30–2.42) | 1.33 (0.67–2.66) | 1.85 (1.23–2.77)** |
| Oligohydramnios | 1.5% (65/4427) | 0.0% (0/158) | 3.4% (7/208) | 2.9% (18/624) | 0.00 (0.00–inf) | 1.16 (0.34–3.88) | 1.81 (0.93–3.53) |
| Induction or augmentation of labor | 36.2% (1604/4427) | 36.1% (57/158) | 43.8% (91/208) | 50.2% (313/624) | 0.78 (0.49–1.25) | 0.96 (0.67–1.37) | 1.52 (1.22–1.91)** |
| NICU admission | 13.4% (372/2782) | 19.3% (16/83) | 12.1% (23/190) | 13.3% (59/442) | 1.77 (0.86–3.64) | 0.92 (0.54–1.57) | 0.97 (0.67–1.40) |
| Preterm birth before 37 weeks of gestation | 9.0% (400/4427) | 13.8% (22/158) | 7.7% (16/208) | 7.5% (47/624) | 1.43 (0.72–2.84) | 0.87 (0.47–1.61) | 0.92 (0.62–1.36) |
| Preterm birth before 34 weeks of gestation | 3.0% (131/4427) | 5.7% (9/158) | 3.4% (7/208) | 3.4% (21/624) | 1.86 (0.64–5.39) | 1.25 (0.48–3.22) | 1.47 (0.81–2.68) |
| Low birth weight (<2,500 g) | 13.1% (582/4427) | 19.6% (31/158) | 13.5% (28/208) | 11.4% (71/624) | 1.18 (0.64–2.18) | 0.90 (0.55–1.49) | 0.84 (0.60–1.17) |
| Very low birth weight (<1,500 g) | 1.8% (80/4427) | 3.2% (5/158) | 1.4% (3/208) | 2.9% (18/624) | 1.54 (0.36–6.65) | 0.81 (0.19–3.45) | 2.03 (1.04–3.98)* |
| SGA | 5.8% (256/4427) | 7.0% (11/158) | 4.8% (10/208) | 4.6% (29/624) | 1.36 (0.61–3.04) | 0.80 (0.38–1.68) | 0.73 (0.45–1.20) |
| LGA | 8.1% (357/4427) | 4.4% (7/158) | 7.2% (15/208) | 11.9% (74/624) | 0.54 (0.19–1.50) | 0.86 (0.45–1.62) | 1.39 (0.98–1.97) |
| FGR | 3.3% (145/4427) | 4.4% (7/158) | 4.8% (10/208) | 2.6% (16/624) | 1.27 (0.39–4.19) | 1.15 (0.45–2.93) | 0.91 (0.47–1.76) |
| Low Apgar score at 5 minutes (<7) | 2.7% (106/3882) | 3.2% (4/126) | 2.0% (4/198) | 4.6% (26/563) | 1.41 (0.42–4.70) | 0.77 (0.23–2.53) | 1.93 (1.11–3.35)* |
| Low Apgar score at 1 minute (<7) | 6.7% (259/3882) | 5.6% (7/126) | 6.1% (12/198) | 9.4% (53/563) | 0.90 (0.36–2.29) | 0.91 (0.45–1.85) | 1.77 (1.22–2.56)** |
| Low umbilical artery pH (<7.1) | 0.6% (25/4427) | 1.3% (2/158) | 0.0% (0/208) | 1.6% (10/624) | 1.93 (0.24–15.35) | — | 3.10 (1.24–7.77)* |

Supplementary Table 5. Incidence of bleeding-related outcomes and adjusted odds ratios in the fresh embryo transfer, OC-FET, and HRC-FET groups, restricted to vaginal deliveries, with the non-ART group as reference (n=3,631)

Values are presented as number (%). Adjusted odds ratios were derived from multivariable logistic regression adjusted for maternal age, pre-pregnancy BMI, and parity, with the non-ART group as reference. **p<0.01.

BMI, body mass index; HRC-FET, hormone replacement cycle frozen embryo transfer; non-ART, non–assisted reproductive technology; OC-FET, ovulatory-cycle frozen embryo transfer; PPH, postpartum hemorrhage.

| Outcome | non-ART (n=3,013) | Fresh ET (n=113) | OC-FET (n=135) | HRC-FET (n=370) | aOR, Fresh ET (vs non-ART) | aOR, OC-FET (vs non-ART) | aOR, HRC-FET (vs non-ART) |
| --- | --- | --- | --- | --- | --- | --- | --- |
| Manual removal of the placenta | 0.7% (20/3013) | 1.8% (2/113) | 2.2% (3/135) | 11.9% (44/370) | 2.30 (0.29–18.27) | 2.67 (0.58–12.29) | 15.95 (7.93–32.10)** |
| PPH | 37.5% (1129/3013) | 37.5% (42/113) | 39.3% (53/135) | 71.9% (266/370) | 1.21 (0.71–2.06) | 0.76 (0.48–1.19) | 3.83 (2.85–5.14)** |
| Blood loss ≥1,500 mL | 1.2% (35/3013) | 0.9% (1/113) | 2.2% (3/135) | 8.1% (30/370) | 1.39 (0.18–10.78) | 2.82 (0.79–10.08) | 8.66 (4.48–16.74)** |
| Blood loss ≥2,000 mL | 0.1% (4/3013) | 0.0% (0/113) | 0.0% (0/135) | 2.7% (10/370) | — | — | — |
| Uterine atony | 9.7% (293/3013) | 8.0% (9/113) | 14.8% (20/135) | 27.8% (103/370) | 1.23 (0.55–2.79) | 1.30 (0.69–2.45) | 3.93 (2.85–5.43)** |

Supplementary Table 6. Incidence of bleeding-related outcomes and adjusted odds ratios in the fresh embryo transfer, OC-FET, and HRC-FET groups, restricted to cesarean deliveries, with the non-ART group as reference (n=1,786)

Values are presented as number (%). Adjusted odds ratios were derived from multivariable logistic regression adjusted for maternal age, pre-pregnancy BMI, and parity, with the non-ART group as reference. *p<0.05, **p<0.01.

BMI, body mass index; HRC-FET, hormone replacement cycle frozen embryo transfer; non-ART, non–assisted reproductive technology; OC-FET, ovulatory-cycle frozen embryo transfer; PPH, postpartum hemorrhage.

| Outcome | non-ART (n=1,414) | Fresh ET (n=45) | OC-FET (n=73) | HRC-FET (n=254) | aOR, Fresh ET (vs non-ART) | aOR, OC-FET (vs non-ART) | aOR, HRC-FET (vs non-ART) |
| --- | --- | --- | --- | --- | --- | --- | --- |
| Manual removal of the placenta | 0.4% (5/1414) | 0.0% (0/45) | 0.0% (0/73) | 5.1% (13/254) | — | — | — |
| PPH | 33.5% (472/1414) | 47.7% (21/45) | 45.2% (33/73) | 41.3% (105/254) | 1.99 (0.90–4.41) | 1.44 (0.84–2.46) | 1.26 (0.88–1.79) |
| Blood loss ≥1,500 mL | 10.5% (148/1414) | 13.6% (6/45) | 16.4% (12/73) | 15.0% (38/254) | 1.60 (0.58–4.44) | 1.03 (0.47–2.28) | 1.19 (0.71–1.97) |
| Blood loss ≥2,000 mL | 4.1% (58/1414) | 9.1% (4/45) | 5.5% (4/73) | 6.7% (17/254) | 3.21 (1.02–10.13)* | 0.30 (0.04–2.25) | 0.97 (0.44–2.17) |
| Uterine atony | 4.9% (69/1414) | 4.5% (2/45) | 6.8% (5/73) | 12.6% (32/254) | 0.66 (0.09–5.06) | 1.16 (0.39–3.44) | 2.27 (1.24–4.16)** |

Supplementary Table 7. Sensitivity analysis of bleeding-related outcomes with additional adjustment for endometriosis or adenomyosis

Values are presented as adjusted odds ratios (95% confidence intervals) derived from multivariable logistic regression for pairwise comparisons among the four groups. Model 1 was adjusted for maternal age, pre-pregnancy BMI, and parity. Model 2 was adjusted for endometriosis or adenomyosis in addition to the Model 1 covariates. Patients with both endometriosis and adenomyosis were counted once. Cases with missing data for the outcome or any covariate were excluded from each analysis. *p<0.05, **p<0.01.

BMI, body mass index; HRC-FET, hormone replacement cycle frozen embryo transfer; non-ART, non–assisted reproductive technology; OC-FET, ovulatory-cycle frozen embryo transfer; PPH, postpartum hemorrhage.

| Comparison | Model | Manual removal of the placenta | PPH | Blood loss ≥1,500 mL | Blood loss ≥2,000 mL | Uterine atony |
| --- | --- | --- | --- | --- | --- | --- |
| Fresh ET vs non-ART | Model 1 | 1.83 (0.24–14.24) | 1.44 (0.93–2.24) | 1.45 (0.61–3.45) | 2.80 (0.95–8.24) | 1.14 (0.54–2.42) |
|  | Model 2 | 1.50 (0.19–11.95) | 1.43 (0.92–2.22) | 1.45 (0.61–3.48) | 2.86 (0.96–8.53) | 1.11 (0.52–2.36) |
| OC-FET vs non-ART | Model 1 | 1.94 (0.43–8.68) | 1.00 (0.71–1.40) | 1.48 (0.77–2.86) | 0.37 (0.05–2.73) | 1.24 (0.72–2.14) |
|  | Model 2 | 1.76 (0.39–7.99) | 0.97 (0.69–1.37) | 1.50 (0.77–2.92) | 0.40 (0.05–2.95) | 1.23 (0.71–2.13) |
| HRC-FET vs non-ART | Model 1 | 14.54 (7.73–27.33)** | 2.33 (1.88–2.89)** | 2.56 (1.76–3.74)** | 2.29 (1.23–4.28)** | 3.23 (2.44–4.27)** |
|  | Model 2 | 14.66 (7.77–27.64)** | 2.32 (1.86–2.87)** | 2.56 (1.75–3.74)** | 2.23 (1.19–4.18)* | 3.27 (2.47–4.33)** |
| OC-FET vs Fresh ET | Model 1 | 1.06 (0.09–11.85) | 0.69 (0.40–1.18) | 1.02 (0.36–2.87) | 0.13 (0.01–1.20) | 1.09 (0.44–2.65) |
|  | Model 2 | 1.04 (0.09–12.08) | 0.64 (0.37–1.11) | 0.95 (0.33–2.74) | 0.12 (0.01–1.17) | 1.07 (0.43–2.65) |
| HRC-FET vs Fresh ET | Model 1 | 7.92 (1.07–58.64)* | 1.61 (1.01–2.58)* | 1.76 (0.73–4.28) | 0.82 (0.27–2.52) | 2.83 (1.32–6.06)** |
|  | Model 2 | 7.99 (1.08–59.18)* | 1.61 (1.00–2.57)* | 1.76 (0.72–4.27) | 0.80 (0.26–2.49) | 2.87 (1.33–6.16)** |
| HRC-FET vs OC-FET | Model 1 | 7.49 (1.78–31.47)** | 2.34 (1.60–3.41)** | 1.73 (0.87–3.44) | 6.24 (0.82–47.52) | 2.60 (1.48–4.58)** |
|  | Model 2 | 7.22 (1.71–30.36)** | 2.40 (1.64–3.51)** | 1.81 (0.90–3.62) | 6.81 (0.89–52.34) | 2.64 (1.50–4.65)** |
